# Lipid Metabolism at Membrane Contacts: Dynamics and Functions Beyond Lipid Homeostasis

**DOI:** 10.3389/fcell.2020.615856

**Published:** 2020-12-23

**Authors:** Jiesi Xu, Xun Huang

**Affiliations:** ^1^State Key Laboratory of Molecular Developmental Biology, Institute of Genetics and Developmental Biology, Innovation Academy for Seed Design, Chinese Academy of Sciences, Beijing, China; ^2^College of Advanced Agricultural Sciences, University of Chinese Academy of Sciences, Beijing, China

**Keywords:** lipid biosynthesis, lipid degradation, membrane contact site, lipid functions, lipid composition

## Abstract

Membrane contact sites (MCSs), regions where the membranes of two organelles are closely apposed, play critical roles in inter-organelle communication, such as lipid trafficking, intracellular signaling, and organelle biogenesis and division. First identified as “fraction X” in the early 90s, MCSs are now widely recognized to facilitate local lipid synthesis and inter-organelle lipid transfer, which are important for maintaining cellular lipid homeostasis. In this review, we discuss lipid metabolism and related cellular and physiological functions in MCSs. We start with the characteristics of lipid synthesis and breakdown at MCSs. Then we focus on proteins involved in lipid synthesis and turnover at these sites. Lastly, we summarize the cellular function of lipid metabolism at MCSs beyond mere lipid homeostasis, including the physiological meaning and relevance of MCSs regarding systemic lipid metabolism. This article is part of an article collection entitled: Coupling and Uncoupling: Dynamic Control of Membrane Contacts.

## Introduction

Compartmentalization is a basic organizational principle of cells. It can be achieved by intracellular membranes, which act as physical barriers to optimize the efficiency of cellular processes that occur within organelles (Aguzzi and Altmeyer, 2016). Lipids are fundamental components of cellular membranes. The lipid composition varies in different organelle membranes and/or subregions/domains within membranes. This heterogeneity of lipid distribution can be achieved by local lipid metabolism or by intracellular lipid trafficking, which delivers lipids from where they are synthesized (in most cases in the endoplasmic reticulum/ER) to their destination membranes and/or membrane domains in both vesicular and non-vesicular pathways.

**FIGURE 1:**
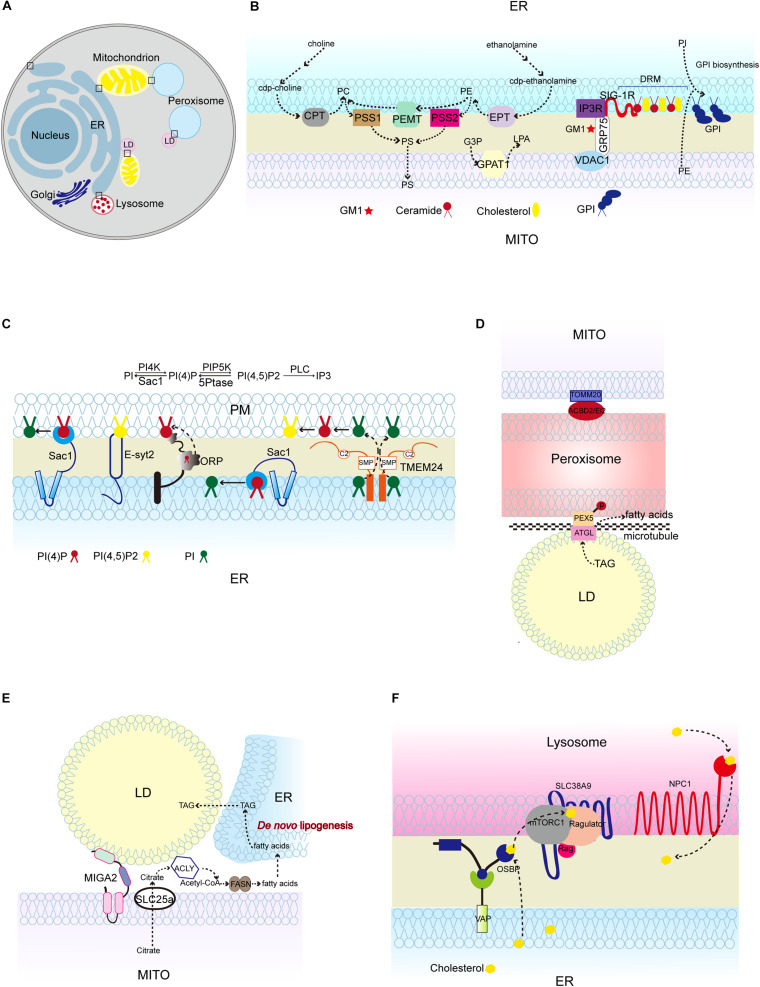
Lipid synthesis and breakdown at MCSs in mammalian cells. **(A)** Schematic illustration of mammalian MCSs. The contact sites discussed in the text are boxed. **(B)** Lipid metabolism at the ER-mitochondrion contacts, highlighting the linked synthesis of PC, PE, and PS; the detergent-resistant membrane (DRM) (domain enriched in ceramide, cholesterol, and associated proteins; and the biosynthesis of GPI using ER-derived PI and mitochondrion-derived PE. **(C)** Lipid metabolism at the ER-PM contacts, showing the Sac1-mediated conversion of phosphatidylinositol 4-phosphate (PI4P) to phosphatidylinositol (4,5)-bisphosphate (PIP2) *in trans* and *in cis*, and TMEM24-mediated PI transport. **(D)** Pex5 and ATGL interaction at peroxisome-LD contacts, and TOMM20 and ACBD2/EI2 interaction at mitochondrion-peroxisome contacts. **(E)** MIGA2-mediated LD-mitochondrion association to supply TAG from mitochondrially derived citrate. **(F)** The activation of mTORC1 by cholesterol delivered from the ER to the lysosome surface by OSBP, and the transport of cholesterol out of the lysosome lumen by NPC1. CPT, CDP-choline-1,2-diacylglycerol choline phosphotransferase (CPT); EPT, CDP-ethanolamine:1,2-diacylglycerol ethanolamine phosphotransferase; PSS1/2, phosphatidylserine synthase; PEMT, phosphatidylethanolamine methyltransferase; GPAT1, glycerophosphate acyltransferases; SIG-1R, Sigma 1R; PC, phosphatidylcholine; PE, phosphatidylethanolamine; PS, phosphatidylserine; G3P, glycerol-3-phosphatase; LPA, lysophosphatidic acid; GM1, GM1-ganglioside; GPI, glycosylphosphatidylinositol; TMEM24, transmembrane protein 24; PEX5, peroxisomal biogenesis factor 5; ATGL, triglyceride lipase; TOMM20, Translocase of outer mitochondrial membrane 20; ACBD2/EI2, acyl-coenzyme A-binding domain; ACLY, ATP citrate lyase; FASN, fatty acid synthase; NPC1, Niemann-Pick disease, type C1; OSBP, oxysterol-binding protein 1.)

**FIGURE 2:**
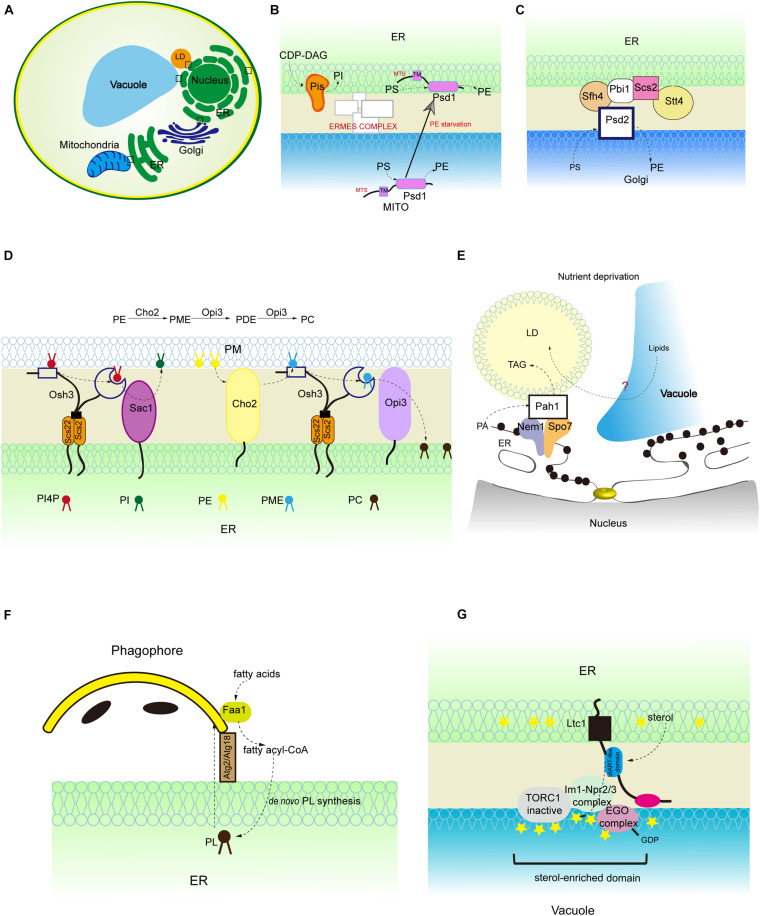
Lipid synthesis and breakdown at MCSs in yeast. **(A)** Schematic illustration of yeast MCSs. The contact sites discussed in the text are boxed. **(B)** The mitochondrial and ER fractions of the PS decarboxylase Psd1, PI synthesis from CDP-DAG at MAMs, and PS transporter:ERMES complex. **(C)** The assembly of ER-Golgi contacts composed of the PS decarboxylase Psd2, Sfh4, the PI4P kinase Stt4, the tether Scs2 that binds Stt4, and Pbi1. **(D)** PI4P turnover at the ER-PM contact, and PC biosynthesis from PE. **(E)** LD-associated Pah1 at the nuclear vacuole junction. **(F)** Local phospholipid synthesis supports phagophore membrane expansion from the ER. **(G)** Concentration of the positive regulator of Torc1 (Ego complex) into sterol-enriched domains at ER-vacuole contacts. PME, phosphatidylmonomethylethanolamine; PDE, phosphatidyldimethylethanolamine.

Membrane contact sites (MCSs) are areas of close apposition between two organelles that mediate non-vesicular lipid trafficking, or between inner and outer membranes of the same organelle, such as mitochondria and chloroplast. It has been known for decades (Scorrano et al., 2019). However, the field of organelle interactions came into the spotlight only when the functional meaning of MCSs was revealed. Vance (1990) described the presence of “fraction X” in mitochondrial preparations, which harbored phospholipid synthetic activity and was later identified as mitochondrial-associated membranes (MAMs) (Vance, 1990). This is the first paper showing that a biochemical activity occurs specifically at contact sites. More recent studies have unveiled the functional significance of MCSs in regulating various cellular processes, such as Ca^2+^ transport, lipid exchange, apoptosis, and organelle biogenesis (Doghman-Bouguerra et al., 2016; Kannan et al., 2017; Wu et al., 2019; Schutter et al., 2020). It is now more evident that such close apposition between organelles facilitates inter-organelle communication and is essential for the structure and function of eukaryotic cells including mammalian cells (Figure 1) and yeast (Figure 2).

Here, we will appraise the findings about the molecular basis, cellular functions, and physiological and pathological implications of lipid metabolism at MCSs. We will not focus on the molecular mechanism of lipid exchange at membrane contact sites, since many excellent reviews have touched on this subject already (Lahiri et al., 2015; Phillips and Voeltz, 2016; Muallem et al., 2017; Balla et al., 2019, 2020; Tamura et al., 2019; Prinz et al., 2020). We will discuss: (1) the characteristics of lipid synthesis and breakdown at MCSs, (2) proteins involved in lipid metabolism at MCSs, and (3) lipid function at MCSs beyond simple lipid homeostasis.

## The Characteristics of Lipid Synthesis and Breakdown at MCSs-Proteins and Lipids

There is growing evidence that intimate physical contacts between the ER membrane and membranes of other organelles play major roles in lipid metabolism, including synthesis, breakdown, and transport (Scorrano et al., 2019). Mass spectrometry reveals that many proteins involved in lipid metabolism are detected in the MCS fractions (Sala-Vila et al., 2016; Ma et al., 2017; Wang et al., 2018). The MAM proteome has been analyzed in various cell lines and mouse tissues (Sala-Vila et al., 2016; Cho et al., 2017; Hung et al., 2017; Ma et al., 2017; Wang et al., 2018; Kwak et al., 2020). For example, using sequential centrifugation to isolate pure MAMs followed by mass spectrometry, over 1000 MAM proteins have been identified (Sala-Vila et al., 2016; Ma et al., 2017; Wang et al., 2018). It has been shown that approximately 10% of the MAM proteins from mouse liver are involved in lipid metabolism, including biosynthesis of cholesterol, fatty acids, steroids and phospholipids, and catabolism of fatty acids (Sala-Vila et al., 2016). Specific proteins at MCSs discussed in this review are listed in Table 1.

**TABLE 1:** Proteins involved in lipid metabolism at MCSs.

**Protein**	**Related membrane contacts**	**System**	**References**
Acyl-CoA synthetase long chain family member 1 (ACSL1)	MAM	mammalian cells	Hung et al., 2017
Carnitine palmitoyltransferase **l**a (CPTla)	MAM	mammalian cells	Hung et al., 2017
Glycerol kinase (GK)	MAM	mammalian cells	Hung et al., 2017
IP3R-GRP75-VDAC1 complex	MAM	mammalian cells	Poston et al., 2011
Sigma-1 receptor (Sigma 1R)	MAM	mammalian cells	Hayashi and Fujimoto, 2010
Sac1 PI phosphatase	ER-PM	mammalian cells	Dickson et al., 2016
Transmembrane protein 24 (TMEM24)	ER-PM	mammalian cells	Lees et al., 2017
Sorting nexin 14 (Snx14)	ER-LD	mammalian cells	Datta et al., 2019
Oxysterol binding protein (OSBP)	ER-lysosome	mammalian cells	Lim et al., 2019
Mitoguardin 2 (MIGA2)	mitochondrion-LD	mammalian cells	Freyre et al., 2019
Adipose triglyceride lipase (ATGL)	peroxisome-LD	mammalian cells	Kong et al., 2020
ACBD2/ECI2 isoform A	peroxisome-mitochondrion	mammalian cells	Fan et al., 2016
Cholinephosphotransferase (CPT)	MAM	rat liver	Vance, 1990
Ethanolaminephosphotransferase (EPT)	MAM	rat liver	Vance, 1990
Glycerophosphate acyltransferase 1 (GPAT1)	MAV	rat liver	Pellon-Malson et al., 2007
Mitofusion2 (MFN2)	MAM	mouse liver	Sebastian et al., 2012; Hernandez-Alvarez et al., 2019
PE methyltransferase (PEMT)	MAM	rat liver	Cui et al., 1993
PS synthases PSS1/2	MAM	rat liver	Vance, 1990
PS decarboxylase1 (Psd1)	MAM	yeast	Wang et al., 2020
PI synthase (Pis)	MAM, PAM	yeast	Gaigg et al., 1995; Pichler et al., 2001
Oxysterol-binding homology 3 (Osh3)	ER-PM	yeast	Stefan et al., 2011
Opi3	ER-PM	yeast	Tavassoli et al., 2013
Ltcl	ER-Vacuole	yeast	Murley et al., 2017
Faal	ER-autophagosome	yeast	Schutter et al., 2020
PS decarboxylase2 (Psd2)	ER-Golgi	yeast	Friedman et al., 2018
Pahl	NVJ associated LD	yeast	Karanasios et al., 2013
Mdml	NVJ associated LD	yeast	Hariri et al., 2018

In recent years, the adoption of an alternative proteomic approach known as proximity-based labeling has advanced the mapping of MCS proteins (Cho et al., 2017; Hung et al., 2017; Antonicka et al., 2020; Kwak et al., 2020). In this approach, a bait protein is endowed with biotinylating activity via fusion with peroxidase (APEX), horseradish peroxidase (HRP), or promiscuous biotin ligase (pBirA); prey proteins near (<10 to 20 nm) the bait protein, or cellular regions enriched in the bait protein, are biotinylated and can then be purified and analyzed (Hung et al., 2017; Antonicka et al., 2020). Several studies have used this method to identify the MAM proteome, and they acquired a much smaller number of MAM proteins compared to conventional fractionation-based methods. One study demonstrated that proteins involved in triglyceride (TAG) synthesis [acyl-coA synthetase long chain family member 1 (ACSL1) and putative glycerol kinase 3 (GK3P) and fatty acid oxidation (carnitine palmitoyltransferase 1a (CPT1a)] are enriched in the MAM fraction of cells (Hung et al., 2017). However, this type of method also has a number of limitations, such as inducing changes in protein localization (toward or away from the membrane) in response to stimuli that are applied by this method, and failing to detect well-known MCS-resident proteins (Kwak et al., 2020).

Although certain lipid synthetic and catabolic enzymes have been identified at the contact sites, the immediate consequence of these enrichments, in particular on the lipid composition of membrane contacts, has scarcely been explored. This is probably due to the difficulty in separation and purification of intracellular membrane without contamination from other membranes (Schuiki et al., 2010; Horvath and Daum, 2013). Thus far, only three studies have analyzed the phospholipid composition of MCSs (Fischl and Carman, 1983; Vance, 1990; Pichler et al., 2001). In rat liver, the molar ratio of phosphatidylcholine (PC)/phosphatidylethanolamine (PE) of MAMs resembles that of the ER rather than the mitochondria (Vance, 1990), which suggests that the properties of MAMs are more similar to the ER in terms of biosynthesis of phospholipids (Vance, 1990). In yeast, analysis of the phospholipid composition of MAMs has revealed that the MAMs have a significantly higher phosphatidylinositol (PI) content and a lower phosphatidic acid (PA) content as compared to mitochondrial and other microsomal membranes (Gaigg et al., 1995). This is probably due to the fact that the highest level of PI synthase (Pis1) activity is found in the MAM fraction (Fischl and Carman, 1983; Gaigg et al., 1995). The lipid composition of ER-plasma membrane (PM) contacts is also more similar to the ER in yeast, with higher levels of PC, PE, and PI and a lower amount of phosphatidylserine (PS) compared to the PM (Pichler et al., 2001).

Similar to the PM, MAMs also contain microdomains, named lipid rafts. Lipid rafts are cholesterol and sphingolipids-rich microdomains in PM. The detergent-resistant lipid rafts in the MAMs from mammalian cells are rich in lipids, such as cholesterol, ceramides and glycosphingolipids, and proteins that are components of MAM-localized Ca^2+^ signaling complexes, such as sigma-1 receptor (Sigma 1R), IP3R, GRP75, and VDAC1 (Hayashi and Fujimoto, 2010; Poston et al., 2011). Of note, the lipid-protein interactions at this specific region play crucial roles in executing both cellular processes and organelle biogenesis. For instance, ceramides from the detergent-resistant membranes of MAMs are physically associated with Sigma 1R and anchor it to the MAMs (Hayashi and Fujimoto, 2010). Sigma 1R stabilizes IP3R3 at MAMs, therefore favoring Ca^2+^ transfer from the ER to the mitochondria (Hayashi and Su, 2007; Figures 1A,B). In mouse brain, GM1-ganglioside (GM1), one of the sialic acid-containing glycosphingolipids (GSLs), physically interacts with phosphorylated form of IP3R in glycosphingolipid-enriched microdomain (GEM) fractions of MAMs and influences Ca^2+^-mediated apoptotic signaling (Sano et al., 2009; Figure 1B). Ganglioside (GD3) interacts with AMBRA1 and WIPI1, both of which are core-initiator proteins responsible for autophagosome formation (Garofalo et al., 2016).

The enrichment of a set of functionally linked lipid biosynthetic enzymes at MCSs also extends our understanding of the lipid composition at MCSs. For example, mammalian MAMs contain the PS synthases PSS1/2, which convert PE and PC to PS, PE methyltransferase (PEMT) which converts PE to PC, and PS decarboxylase (PISD) which converts PS to PE. The linked synthesis of PS, PE, and PC segregates the pools of PS and PS-derived phospholipids from the bulk of the ER phospholipids (Vance, 1990). The segregation of these specific chemical reactions may increase the reaction efficiency and restrict the dissemination of reaction products, or may serve special functions. For instance, the cell uses the pool of PS-derived phospholipids for lipoprotein assembly (Vance, 1990).

Each subcellular compartment of the cell has a specific set of membrane lipids. PC and PE are the most abundant phospholipids in the membranes of mammalian and yeast cells (Horvath and Daum, 2013). PS is highly enriched in the PM of these cells (Horvath and Daum, 2013). PI is more enriched in the Golgi apparatus than in other subcellular compartments (Horvath and Daum, 2013). Sphingolipids are enriched in lysosomes (Horvath and Daum, 2013). To maintain the distinct membrane features and lipid composition of each subcellular compartment, coordinated regulation of lipid synthesis, degradation, and transport is required. The identification of lipid metabolic enzymes at MCSs and the lipid composition of MCSs lays the foundation of our understanding of lipid metabolism at MCSs. In the next part, we will discuss in detail the individual enzymes involved in lipid synthesis and breakdown at MCSs.

## Lipid Metabolism at the Membrane Contacts

### Phospholipid Synthesis and Breakdown at Membrane Contacts

Several lines of evidence suggest that non-vesicular lipid transport intersects with lipid biosynthetic and regulatory pathways at MCSs (Fernandez-Murray and McMaster, 2016; Phillips and Voeltz, 2016; Balla et al., 2019; Figures 1A,  2A). In this part, we review studies of phospholipid metabolism at MCSs, with the emphasis on phospholipid biosynthesis and breakdown in mammalian system and yeast.

## PS

Phosphatidylserine is transported from the ER to mitochondria and decarboxylated to synthesize PE, and PE is transferred in the reverse direction from mitochondria to the ER. The function of MAMs in PS import into mitochondria has been extensively studied in mammalian cells and yeast (Vance, 1990; Voelker, 1990; Gaigg et al., 1995; Shiao et al., 1995; Achleitner et al., 1999; Nguyen et al., 2012; Lahiri et al., 2014; Hernandez-Alvarez et al., 2019; Petrungaro and Kornmann, 2019). The best-studied ER-mitochondria tether that is responsible for PS transport is yeast ER-mitochondria encounter structure (ERMES) complex (Kawano et al., 2018; Petrungaro and Kornmann, 2019; Figure 2B). In addition to facilitating PS transport, MAMs also contains phospholipid synthetic activity. An excellent study performed by J. E. Vance demonstrated that the activities of phospholipid biosynthetic enzymes such as PS synthase (PSS), cholinephosphotransferase (CPT) and ethanolaminephosphotransferase (EPT) are present in MAMs (Vance, 1990) (Figure 1B). Later on, her group reported that both full-length PSS1 and PSS2 are located almost exclusively at MAMs and are largely excluded from the bulk of the ER (Stone and Vance, 2000; Figure 1B). The presence of phospholipid biosynthetic enzymes at the MAMs raises the following question: is the unique subcellular localization of these enzymes functionally significant in the regulation of metabolic processes? In fact, the newly synthesized PS is more readily transported from the ER to the mitochondria than the preexisting PS (Vance, 1991; Achleitner et al., 1995). Similarly, the newly synthesized PE is preferred for translocation from the mitochondria to the ER (Vance, 1991; Achleitner et al., 1995). Recently, Prinz et al. (2020) fused *E. coli* PS synthase with yeast Mmm1, which is specifically located to the MAMs, or with yeast Sec63, which is evenly distributed in all portions of the ER (Kannan et al., 2017). Their results showed that enrichment of PS synthase at the MAMs promotes more efficient PS transport than when PS synthase is located evenly in the ER (Kannan et al., 2017). This suggests that a MAM-localized phospholipid synthetic enzyme can increase phospholipid transport.

In mammalian cells, ER-derived PS is rapidly converted to PE by decarboxylation and this is thought to take place in mitochondria only. However, yeast has two PS decarboxylases: mitochondrial Psd1 and the *trans-*Golgi network/endosomal Psd2 (Wang et al., 2020). Psd2 is proposed to engage with MCSs for PS decarboxylation (Wang et al., 2020). Several proteins are known to be required in conjunction with Psd2 for PS transport to occur, such as the sec14-like phosphatidylinositol transfer protein (PITP) Sfh4, the phosphatidylinositol 4-kinase (PI4K) Stt4, the tether Scs2, and an uncharacterized protein Pbi1 (Wang et al., 2020; Figure 2C). A recent study demonstrated that Sfh4 affects Psd2 activity through direct physical interaction with Psd2 and the functional effect of Sfh4 is independent of its PI-binding/exchange activity (Wang et al., 2020). This study challenges the general view of PITP as a PI transfer protein. Although Psd1 is considered as an inner mitochondrial membrane-anchored protein, a recent study showed that Psd1 has dual ER and mitochondrial localization with its transmembrane domain necessary and sufficient for its ER localization (Friedman et al., 2018; Figure 2B). Furthermore, the mitochondrial fraction of Psd1 is required for normal mitochondrial function and the ER-localized fraction of Psd1 is required for normal cellular PE homeostasis (Friedman et al., 2018). This finding implies that the mitochondrial-derived PE generated by mitochondrial Psd1 is not robust enough to provide cells with a sufficient PE pool. Thus, the different organelle-associated domains of a protein may play distinct but essential roles in organelle function.

## PI and PI4P

Phosphatidylinositol is synthesized in the ER and phosphorylated to PtdIns 4-phosphate (PI4P) at the PM and Golgi and PtdIns 4,5-biphosphate (PIP2) at the PM (Agranoff et al., 1958; Gaigg et al., 1995; Figure 1C). Mammalian phosphatidylinositol synthase (PIS), which catalyzes PI formation using CDP-DAG, is found at the ER. Interestingly, PIS has been detected in a highly mobile membrane compartment, which originates from the ER and provides PI to cellular membranes in mammalian cells (Kim et al., 2011). In addition, the autophagy initiation complex is located to the PIS-enriched ER subdomains of mammalian cells (Nishimura et al., 2017). In yeast, the specific activity of phosphatidylinositol synthase (Pis) is significantly higher in the MAM fraction than in the ER fractions (Gaigg et al., 1995; Figure 2B). The PI level in the MAMs is almost three times higher than that in ER fractions (Gaigg et al., 1995). The biosynthesis of PI is also enriched in the ER-associated plasma membrane (PAM) in yeast (Pichler et al., 2001).

PI4P, derived from PI by PI kinase, is an essential signaling molecule at the PM and Golgi with functions in signal transduction, lipid metabolism, and membrane trafficking (D’Angelo et al., 2008). Sac1 PI phosphatase is an important regulator of PI4P turnover and is located to the ER and Golgi (Nemoto et al., 2000; Foti et al., 2001; Faulhammer et al., 2007). *sac1* mutant yeast cells accumulate PI4P at the PM (Baird et al., 2008). Since Sac1 is not known to traffic to the PM, there must be factors that link Sac1 activity to PI4P at the PM (Stefan et al., 2011). Oxysterol-binding homology 3 (Osh3), a conserved pleckstrin-homology (PH) domain-containing protein, is identified as linking Sac1 activity to PI4P homeostasis at the PM (Stefan et al., 2011). PI4P binds to the Osh3 PH domain and activates Osh3 at the ER-PM contact sites (Stefan et al., 2011). The association of PI4P with Osh3 facilitates the interaction between ORD, a lipid transfer domain in Osh3, and the downstream target protein Sac1, thus stimulating Sac1 PI phosphatase activity (Stefan et al., 2011; Figure 2D). Therefore, Osh proteins can act as sensors of PI4P at the PM and activators of Sac1 phosphatase at the ER. Although these findings support the notion that Sac1 controls the PI4P level at the PM *in trans*, some evidence suggests that it acts *in cis* (i.e., in the same membrane) (Mesmin et al., 2013). In fact, Sac1 dephosphorylates PI4P at the ER and creates a PI4P gradient. This process is accompanied by counter transport of cholesterol or PS by oxysterol-binding protein (OSBP) and Osh6, respectively, and is conserved in yeast and mammalian systems (von Filseck et al., 2015; Mesmin et al., 2017). In mammalian cells, Sac1 is reported to be located at the ER-PM junctions (Dickson et al., 2016). Depletion of PI(4,5)P2, the product from phosphorylation of PI4P, at the PM reduces the amount of Sac1 in contact with the PM, thus limiting PI4P dephosphorylation through a feedback mechanism (Dickson et al., 2016; Figure 1C). The above findings about the functions of lipid transfer proteins at the ER-PM contacts shed light on their roles in maintaining contact structures and the PM lipid composition. Indeed, elimination of lipid transfer proteins causes dysregulation of phospholipid biosynthesis and sterol transfer, which negatively impacts PM organization (Quon et al., 2018).

## PA

Phosphatidic acid can be derived from lipid precursor: glycerol 3-phosphate (G3P). In this process, G3P is acylated by glycerophosphate acyltransferases (GPATs) to form lyso-PA which is further converted to PA by 1-acylglycerol 3-phosphate acyltransferases (AGPATs) (Gonzalez-Baro et al., 2007; Takeuchi and Reue, 2009). Thus far, four mammalian GPAT proteins have been identified. There are three N-ethylmaleimide (NEM)-sensitive microsomal and mitochondrial GPATs (GPAT2-4) and one NEM-resistant mitochondrial GPAT1 (Wang et al., 2007; Nagle et al., 2008). Because the enzymes that catalyze the final steps of TAG synthesis are localized to the ER, the mitochondrial localization of GPAT1 is unexpected. A study has shown that GPAT1 is highly enriched in the mitochondrial-associated vesicle (MAV) fraction, which is obtained from sedimentation of the upper band from Percoll density gradient centrifugation of crude mitochondria (Pellon-Malson et al., 2007; Figure 1B). MAVs share characteristics with both MAMs and crude mitochondrial fraction, which contains mitochondrial and MAM fractions. Many marker proteins present in above fractions are also recovered in the MAV fraction. The MAV fraction contains large vesicles, as viewed by electron microscopy (Pellon-Malson et al., 2007). Although the protein level of GPAT1 is highly enriched in this MAV fraction, GPAT1 activity is most enriched in pure mitochondria (Pellon-Malson et al., 2007). This suggests that GPAT1 is largely inactive in the MAV fraction. The discrepancy between GPAT1 protein expression and activity in the subcellular fraction suggests the possibility that GPAT1 in the MAV fraction may have novel roles beyond its enzymatic activity, and, as such, it has been postulated that GPAT1 from the MAV fraction is important for transporting its product, lyso-PA, from the mitochondria to the ER (Pellon-Malson et al., 2007).

## PC

Phosphatidylcholine is the most abundant phospholipid in mammalian cells. PC is synthesized via either the CDP-choline pathway or the methylation of PE (Horvath and Daum, 2013). Liver-specific PEMT, which converts PE to PC, is specifically located at the MAMs (Cui et al., 1993; Figure 1B). Although PEMT is highly enriched in the MAMs, the PE methyltransferase activity in the MAMs is comparable with that in the ER, which indicates that other ER-localized enzymes may also have PE methyltransferase activity (Vance and Vance, 1988; Cui et al., 1993; Rusinol et al., 1994). In yeast, methylation of PE is the primary pathway for the biosynthesis of PC when cells are grown in the absence of choline, whereas the CDP-choline pathway is an auxiliary route since it requires exogenous choline (McDonough et al., 1995). Unlike the mammalian PEMT, which catalyzes all three transmethylation steps to form PC, yeast has two PEMT enzymes, designated Cho2 and Opi3, which catalyze the first and the last two consecutive transmethylation steps, respectively (Cui et al., 1993; Figure 2D). Of interest, a study showed that the ER-PM contacts are required for PC synthesis through the methylation of PE (Tavassoli et al., 2013). *SCS2* and *ICE2*, two ER-localized proteins, play important roles in ER biogenesis and the structure of ER-PM contacts (Tavassoli et al., 2013). Δ*scs2*Δ*ice2* mutant yeast exhibited disrupted ER-PM contacts, growth defects and reduced PC synthesis (Tavassoli et al., 2013). The reduced PC synthesis is due to the loss of function of Opi3 (Tavassoli et al., 2013). With disrupted ER-PM contacts, the access of lipid substrates such as phosphatidylmonomethylethanolamine (PME) and phosphatidyldimethylethanolamine (PDE) to Opi3 is compromised (Figure 2D). In addition, similar to the Sac1-Osh3 regulatory relationship at the ER-PM contacts, Osh3 also regulates Opi3 and facilitates its PC synthetic activity at these contacts (Stefan et al., 2011; Tavassoli et al., 2013; Figure 2D). The precise regulation of PC biosynthesis at the ER-PM contacts is crucial, because in yeast, Opi3 controls the ratio of PE:PC at the PM and decreased Opi3 activity results in an increased PE:PC ratio, therefore destabilizing the PM bilayer (Schueller et al., 2007).

Compelling evidence suggests that many phospholipid biosynthetic enzymes are enriched at MCSs. It is a paradox that their enzymatic activities are not always enriched at MCSs, which suggests that non-enzymatic roles/domains of these proteins may be important for their function at MCSs. The contact sites provide a confined environment for segregating phospholipid biosynthetic enzymes. Therefore, the pool of lipids generated by this segregation may serve special purposes, such as transport to other organelles or involvement in lipoprotein synthesis. Furthermore, the contact sites can spatially regulate the accessibility of lipid substrates to their catalytic enzymes. Therefore, the fine regulation of chemical reactions can be accomplished at MCSs.

### Neutral Lipid Synthesis and Degradation at Membrane Contacts

The ER synthesizes phospholipids for membrane growth and cell proliferation, and TAG to store energy in lipid droplets (LDs). LD biogenesis is generally considered to occur at the ER. In some cell types, LDs appear inside the nucleus (Layerenza et al., 2013; Uzbekov and Roingeard, 2013; Ohsaki et al., 2016). Yeast phosphatidate phosphatase, Pah1, catalyzes the conversion of PA to DAG, which channels PA toward TAG storage but away from phospholipid synthesis for membrane biogenesis and growth. Pah1 lacks transmembrane domains and requires translocation onto membranes to become functional (Karanasios et al., 2013). It has been shown that the acidic tail of Pah1 is required for both LD and nuclear membrane recruitment (Karanasios et al., 2013). It is likely that membrane-bound Pah1 and its regulation of lipid and membrane biogenesis are key metabolic adaptations when the cell requires drastic membrane remodeling (Karanasios et al., 2013). Indeed, during glucose exhaustion in yeast, Pah1 is targeted transiently to the nuclear membrane domain that contacts the vacuole, named the nuclear vacuole junction (NVJ) (Barbosa et al., 2015; Figure 2E). Subsequently, Pah1 is concentrated in two nuclear membrane puncta flanking the NVJ that are in contact with LDs (Barbosa et al., 2015; Figure 2E). The biological significance of this concentration of Pah1 and the associated LDs at the NVJ flanked by the nuclear envelope is not completely clear. Given that in nutrient-rich conditions in yeast, phospholipid synthesis is predominant, whereas during glucose exhaustion, lipid precursors are redirected to TAG storage, it is possible that Pah1 facilitates NVJ-mediated degradation of the nuclear membrane and LD biogenesis, both of which are lipid recycling processes during glucose exhaustion. Recent evidence suggests that yeast Mdm1 and its human homolog: sorting nexin protein (Snx14) are localized to NVJ-associated LD (ER-LD contact in mammalian cells) and regulate NVJ-associated LD production (Schuldiner and Bohnert, 2017; Eisenberg-Bord et al., 2018; Hariri et al., 2018; Datta et al., 2019).

It is clear that LD-organelle contacts are regulated by nutritional status. In mammalian system, the stored TAG undergoes lipolysis in adipocytes to release fatty acids and glycerol in response to starvation, and this process is mediated by TAG hydrolases including adipose triglyceride lipase (ATGL) and hormone-sensitive lipase (HSL). The released fatty acids are further oxidized in mitochondria or peroxisomes. Lipolysis coupled with fatty acid oxidation supplies energy during starvation. An interesting study revealed that fasting promotes the interaction between peroxisomes and LDs (Kong et al., 2020). Upon fasting, peroxisomal biogenesis factor 5 (PEX5) mediates the recruitment of ATGL at peroxisome-LD contact sites (Kong et al., 2020). Lipolysis is compromised if peroxisome-LD contacts are disrupted (Kong et al., 2020; Figure 1D). This study provides a clear example of how cells respond to environmental stress such as nutrient depletion by modulating organelle contacts (Zaman et al., 2008). In fact, cells can initiate various adaptations in response to cellular stress. For instance, during prolonged starvation in yeast, ER-mitochondria contact sites are lost, concomitant with sequestration of both cytosolic and ER lipid biosynthetic enzymes into deposits (Suresh et al., 2015). These two processes are considered as an adaptive response for yeast cells to regulate lipid flux when the supply of nutrients is limited (Suresh et al., 2015). The underlying mechanism is not completely clear. It might be that sequestration of enzymes permits regulation of lipid homeostasis without affecting the enzymatic activities and enables cells to quickly alter their lipid flux by simply relocalizing their enzymes when the nutritional status is favorable (Suresh et al., 2015).

Besides the aforementioned peroxisome-LD associations during starvation in cells, mitochondrion-LD associations have also been found in white and brown adipocytes (Benador et al., 2018). It is conceivable that there are factors that regulate mitochondrion-LD contacts. Through proteomic analysis of adipocyte LDs, a mitochondrial outer membrane protein, Mitoguardin 2 (MIGA2), was found to be associated with LDs in adipocytes or oleic acid-treated COS7 cells (Freyre et al., 2019; Figure 1E). MIGA2 is not only found at mitochondrion-LD contacts; it is also located to the ER, where *de novo* lipogenesis mainly takes place (Freyre et al., 2019; Figure 1E). It has been shown that MIGA2 promotes lipogenesis from non-lipid precursors such as citrate in the mitochondria, possibly leading to positive feedback to the adipogenic transcriptional program and driving adipogenesis and LD formation forward (Freyre et al., 2019). These results coincide with the finding that LD-associated mitochondria support LD expansion by increasing TAG synthesis (Benador et al., 2018).

### Synthesis and Breakdown of Other Lipids at Contact Sites

In mammalian system, glycosylphosphatidylinositol (GPI) biosynthetic reactions are largely confined to MAMs (Figure 1B). GPIs are important for anchoring proteins to the cell membranes (Vidugiriene et al., 1999). It is likely that the localization of GPI biosynthetic activity at MAMs may allow the biosynthetic enzyme more accessibility to its substrate PE, which is mainly derived from decarboxylation of PS in mitochondria (Vidugiriene et al., 1999). Peroxisomes and mitochondria share some similarities in terms of their synergistic functioning in the metabolism of fatty acids, reactive oxygen species, and steroid biosynthesis (Fan et al., 2016). It has been demonstrated that peroxisomes are physically associated with mitochondria and that Pex34, a peroxisomal membrane protein, and Fzo1, the yeast mitofusion, serve as tethers of peroxisome-mitochondria contact (Fan et al., 2016; Shai et al., 2018). A family of acyl-CoA-binding domain (ACBD)-containing proteins regulates steroid biosynthesis in both peroxisomes and mitochondria (Figure 1D). Recent findings suggest that peroxisomal ACBD2/ECI2 isoform A, generated by alternative splicing, is also located to the mitochondria and mediates peroxisome-mitochondrion contact and steroid biosynthesis (Fan et al., 2016).

The autophagosome mediates the degradation of cytoplasmic materials by macroautophagy and is formed in close proximity to the ER (Zhao and Zhang, 2019). Autophagosome formation involves the nucleation of a single-membrane phagophore and its further expansion and closure of its membrane (Mari et al., 2010; Shima et al., 2019). This raises a question: what membranes or processes sustain autophagic membrane formation? It is considered that many organelles, such as ER, Golgi, endosomes, mitochondria, and plasma membrane, contribute to the formation of autophagosomes (Axe et al., 2008; Geng et al., 2010; Hailey et al., 2010; Puri et al., 2013; Nishimura et al., 2017). However, a study demonstrated that *de novo* phospholipid synthesis contributes to autophagosome membrane formation in yeast, which suggests a unique mechanism (Schutter et al., 2020). It has been shown that the long-chain acyl-CoA synthetase (Faa1), which catalyzes the formation of fatty acyl-CoA, is localized to nucleated phagophores. Faa1 channels activated FAs locally into *de novo* phospholipid synthesis at the ER, which forms stable contacts with nascent autophagosomes (Schutter et al., 2020; Figure 2F). Furthermore, the newly synthesized phospholipids at the ER promote the assembly and expansion of the phagophore membrane into an autophagosome (Figure 2F). The concentrated Faa1 activity specifically on nucleated phagophores allows spatiotemporal compartmentalization of *de novo* phospholipid synthesis, which readily facilitates autophagic membrane expansion under starvation conditions. This notion is conceptually similar to the idea discussed above, that the newly synthesized lipids at the contact sites support the local lipid flux between ER and tethered organelles (Kannan et al., 2017). Therefore, the fine spatial segregation of molecular components permits efficient organelle communication and is critical for cellular homeostasis.

In sum, based on the presence of many lipid biosynthetic enzymes at MCSs and their physiological significances in cellular processes, we may reconsider MCSs as being involved in both organizing lipid synthesis and facilitating intermembrane lipid transport. Newly synthesized lipids at MCSs may have distinct functions beyond simple lipid homeostasis, which will be discussed in the next part.

## Lipid Functions at Contact Sites Beyond Simple Lipid Homeostasis

### Distinct Functions of Lipids at MCSs

Lipids at contact sites are critical in maintaining lipid homeostasis and membrane organization. Furthermore, some lipids at membrane contact sites are capable of regulating enzyme activity or signal transduction. Here, we will review the findings about lipid function at contact sites beyond simple lipid metabolism.

As mentioned at the start of this review, compartmentalization is a key determinant of cellular function and biology. One type of compartmentalization is the specialized membrane domains that exist within membrane lipid bilayers (Rai et al., 2016). Membrane contact sites persist during harsh mechanical and chemical separation methods (Vance, 1990). It is possible that specific lipids and proteins are assembled and organized into membrane domains and tether contact sites which are of biophysical and physiological importance in living cells (King et al., 2020). Certain lipid species such as sterols and sphingolipids have greater propensity for membrane domain biogenesis (Harder and Simons, 1997; Simons and Ikonen, 1997). In addition, MCS-resident proteins can also facilitate membrane domain compartment formation. For instance, Osh proteins at ER-PM contact sites create a nanoscale membrane environment that facilitates the synergistic transport of unsaturated PS and sterol and stimulates phosphatidylinositol-4-phosphate 5-kinase (PIP5K) activity, thus affecting PIP2 generation and its related cellular events at the PM (Nishimura et al., 2019). Another example of how small-scale lipid organization controls an enzyme activity or signaling events is provided by the yeast sterol transport protein, Ltc1. It is found at ER-vacuole contact sites and facilitates the partitioning and concentration of the EGO complex, a positive regulator of TORC1, into sterol-enriched domains, thus inhibiting TORC1 activity during stress conditions in yeast (Murley et al., 2015, 2017; Figure 2G). These findings suggest that lipids together with membrane-associated proteins can be concentrated into membrane domains at MCSs and enable localized signal transduction.

In addition to being regulated by sterol-enriched membrane domains, mTORC1 activity can be activated by cholesterol on the surface of lysosomes in mammalian cells (Castellano et al., 2017). A study showed that oxysterol binding protein (OSBP), which is located to the ER-lysosome contacts, ensures ER-to-lysosome cholesterol transfer and mTORC1 activation (Lim et al., 2019; Figure 1F). Cholesterol from the ER-lysosome contact sites directly interacts with mTORC1 scaffolding proteins, leading to mTORC1 activation on the lysosomal surface (Lim et al., 2019; Figure 1F). NPC1 handles LDL-derived cholesterol and transfers cholesterol from the lysosomal lumen to other acceptor membranes (Gong et al., 2016; Figure 1F). NPC1-deficient cells have increased accumulation of cholesterol in lysosomes and hyperactive mTORC1. Inhibition of OSBP attenuates hyperactivity of mTORC1 signaling in NPC1-deficient cells by inhibiting the transfer of cholesterol from the ER to the lysosomal surface (Lim et al., 2019). This work uncovered the effect of cholesterol transfer at ER-lysosome contacts on the regulation of mTORC1 activity and shed light on the molecular mechanism underlying the pathogenesis of neurodegenerative diseases caused by inactivation of NPC1.

PIP2 at the PM controls insulin release from pancreatic beta cells (Xie et al., 2016). Transmembrane protein 24 (TMEM24) serves as a tether at ER-PM contact sites and has an SMP domain, which is capable of transporting PI, the precursor of PIP2, from its site of synthesis in the ER to the PM during glucose-induced insulin secretion (Lees et al., 2017; Figure 1C). TMEM24 also plays a critical role in calcium pulsatility, likely by replenishing PIP2 pools at the PM, which positively regulate IP3 receptors and the PM ion channels that control calcium influx by generating IP3 (Lees et al., 2017). This finding illuminates the elegant mechanism underlying the regulatory effect of an organelle contact-resident lipid-transfer protein on PI pools, Ca^2+^ oscillation and insulin secretion in beta cells (Lees et al., 2017). Together, these findings reveal the function of lipids at MCSs in regulating MCS integrity and localized signal transduction.

### Physiological Relevance of MCSs

Growing evidence suggests that MCS function is linked to neurodegenerative diseases, Alzheimer disease and metabolic diseases. Here, we will review the physiological function of MCSs in the development of metabolic diseases (Table 2).

**TABLE 2:** Physiological function of MAMs in metabolic diseases.

**Disease**	**Protein**	**References**
Diabetes	Cyclophilin D (CypD)	Tubbs et al., 2014
Diabetes	MFN2	Sebastian et al., 2012; Shinjo et al., 2017
Diabetes	PDK4	Thoudam et al., 2019
Diabetes	FATE1	Tubbs et al., 2018
Diabetes	CISD2	Chen et al., 2009; Wang et al., 2014
Diabetic heart disease	FUNDC1	Wu et al., 2019
Obesity	IP3R-GRP75-VDAC1 complex, PACS2	Arruda et al., 2014
NAFLD	MFN2	Hernandez-Alvarez et al., 2019
NAFLD	PEMT	Jacobs et al., 2017
NAFLD	PCYTla	Fu et al., 2011
Cardiac hypertrophy	IGF1	Gutierrez et al., 2014

Mitochondrial-associated membranes play crucial roles in regulating a variety of metabolic stresses, including virus infection, ER stress, hypoxia, nutrient deprivation, and excess glucose availability (Simmen and Herrera-Cruz, 2018). Since recent evidence suggests that MAMs are fundamentally important for hormonal and nutrient signaling, MAMs have come into the spotlight of research on metabolic diseases. MAM structure and function have been implicated in insulin sensing and glucose homeostasis in various tissues, such as liver, adipose tissue, and skeletal muscle. In the liver of diet-induced obese and diabetic mice, ER-mitochondrion contacts are increased, thus resulting in mitochondrial Ca^2+^ overload, oxidative stress and dysfunction, and insulin resistance (Arruda et al., 2014). This seems to be a paradox, because in hepatocytes, palmitate treatment reduces ER-mitochondrion contacts and insulin signaling, and induction of MAMs by overexpression of mitofusin 2 (Mfn2) or GRP75 can rescue the palmitate-induced aberrant insulin signaling (Tubbs et al., 2014; Shinjo et al., 2017). Moreover, loss of Mfn2 reduces ER-mitochondrion interactions and causes insulin resistance and altered glucose homeostasis (Sebastian et al., 2012). Accordingly, Tubbs et al. (2014) observed that MAM integrity is disrupted in the liver of obese and diabetic mouse models. In addition, MAM function has been linked to the pathogenesis of non-alcoholic fatty liver disease (NAFLD). For instance, deficiency of some MAM resident proteins involved in phospholipids biosynthesis, such as PEMT and phosphate cytidylyltransferase 1, choline, alpha (Pcyt1a), cause liver damage in mice (Fu et al., 2011; Jacobs et al., 2017). Furthermore, Recent evidence shows that liver-specific deletion of Mfn2 causes defected PS transport between the ER and mitochondria and leads to non-alcoholic fatty liver disease (Hernandez-Alvarez et al., 2019).

In skeletal muscle, it is found that obesity enhances MAM formation (Thoudam et al., 2019). Inactivation of PDK4 reduces MAM formation and improves insulin signaling in obese mice by disrupting the interaction between PDK4 and the IP3R1-GRP75-VDAC1 complex, which regulates Ca^2+^ transport and controls MAM stability (Thoudam et al., 2019). However, contradictory results showed that ER-mitochondrion contacts in skeletal muscle are disrupted in different mouse models of obesity and diabetes (Tubbs et al., 2018). Furthermore, experimental increase of the ER-mitochondrion contacts in human myotubes prevents palmitate-induced aberrant insulin sensitivity (Tubbs et al., 2018). The reason for the discrepancy between these studies is not clear, but is likely related to differences in the experimental systems and/or experimental analysis methods.

In adipose tissue, CDGSH iron sulfur domain 2 (Cisd2)-mediated loss of ER-mitochondrion contacts impairs mitochondrial Ca^2+^ uptake, decreases insulin-stimulated glucose transport and results in mitochondrial dysfunction (Chen et al., 2009; Wang et al., 2014). Furthermore, miscommunication between ER and mitochondria is an essential step in the pathogenesis of cardiac hypertrophy. Treatment of cardiomyocytes with norepinephrine increases the distance between ER and mitochondria and decreases insulin-induced mitochondrial Ca^2+^ uptake, thus resulting in insulin desensitization (Gutierrez et al., 2014). Glucose is identified as a novel regulator of MAMs and it reduces the ER-mitochondrion contacts, induces mitochondrial fission, and impairs mitochondrial respiration in hepatocytes (Theurey et al., 2016). In line with this, disruption of MAM integrity mimics the effects of glucose on mitochondrial dynamics and function (Theurey et al., 2016). Recently, it is suggested that genetic downregulation of FUN14 domain containing 1 (Fundc1) improved mitochondrial function in HG-treated cardiomyocytes (Wu et al., 2019). Based on recent studies, it is clear that MAMs are involved in metabolic diseases. However, studies on this topic are controversial. MAMs may be a target for treating metabolic diseases, but more studies on their physiological role and regulation are required.

## Concluding Remarks

Membrane contact sites permit the speed and spatial confinement that are required for the intricate control of cellular processes and organelle biogenesis. It has been observed that MCSs are resistant to harsh separation methods, probably because of the biophysical properties of their resident membrane proteins and lipids. There is a general view that lipids allow particular proteins in membranes to aggregate, and others to disperse. In fact, lipids and associated membrane proteins can form nanoscale domains at MCSs. Furthermore, it is an emerging concept that the formation of membrane contacts depends on the lateral segregation of lipids into domains, where lipid and protein binding domains recognize and integrate signals between the donor and/or acceptor membranes (van Meer et al., 2008). Therefore, lipid composition is essential for the biophysical, biochemical and physiological properties of MCSs. On the other hand, lipids synthesized at MCSs may serve special functions, such as ready transportation to an organelle, or acting as sensing molecules to transmit signals in transduction pathways, or stimulating local enzymatic activity. Accordingly, understanding the lipid composition of MCSs will be of great value to delineate their function. Currently, a comprehensive lipidomic analysis of MCSs has not been reported.

Another interesting phenomenon is that multiple phospholipid synthetic enzymes are enriched at MCSs. The segregation of these enzymes may allow generation of a local pool of phospholipids to support organelle membrane biogenesis or local signaling. Revealing the functions of distinct pools of lipids *in vivo* will be a great challenge. In addition, the discrepancy between the enriched enzymatic activities and their protein levels at MCSs may suggest that non-enzymatic functions of these proteins exist at contact sites. Furthermore, the regulation of the activity at MCSs may depend on specific actors or different local environment for activity such as lipid microdomains. The presence of these enzymes at MCSs may also permit more efficient access to their lipid substrates, or may generate a gradient of lipids (such as PI4P) between the donor and acceptor membranes to facilitate local lipid transport, or may regulate the phospholipid composition of adjacent organelles (such as PE levels in mitochondria and ER, controlled by Psd1).

In the last decade, MCSs have been implicated in metabolic diseases. However, the studies are sometimes controversial. Although a repertoire of methods has been applied to study MCSs, it still remains challenging to identify the contact sites, due to their transient nature and various abundance in different cell types. The current findings usually use knockout of an MCS-resident protein to study the link between MCSs and metabolic diseases. However, this approach may suffer from bias; for instance, the results may be caused by functions of the proteins other than their role at MCSs. Therefore, determining how lipid metabolism specifically at MCSs directly contributes to the pathogenesis of metabolic diseases will be an important future endeavor.

## Author Contributions

Both authors listed have made a substantial, direct and intellectual contribution to the work, and approved it for publication.

## Conflict of Interest

The authors declare that the research was conducted in the absence of any commercial or financial relationships that could be construed as a potential conflict of interest.

## References

[B1] Achleitner G. Gaigg B. Krasser A. Kainersdorfer E. Kohlwein S. D. Perktold A. (1999). Association between the endoplasmic reticulum and mitochondria of yeast facilitates interorganelle transport of phospholipids through membrane contact. *Eur. J. Biochem.* 264 545–553. 10.1046/j.1432-1327.1999.00658.x 10491102

[B2] Achleitner G. Zweytick D. Trotter P. J. Voelker D. R. Daum G. (1995). Synthesis and intracellular-transport of aminoglycerophospholipids in permeabilized cells of the yeast, *Saccharomyces cerevisiae*. *J. Biol. Chem.* 270 29836–29842. 10.1074/jbc.270.50.29836 8530379

[B3] Agranoff B. W. Bradley R. M. Brady R. O. (1958). The enzymatic synthesis of inositol phosphatide. *J. Biol. Chem.* 233 1077–1083. 13598735

[B4] Aguzzi A. Altmeyer M. (2016). Phase separation: linking cellular compartmentalization to disease. *Trends Cell Biol.* 26 547–558. 10.1016/j.tcb.2016.03.004 27051975

[B5] Antonicka H. Lin Z. Y. Janer A. Aaltonen M. J. Weraarpachai W. Gingras A. C. (2020). A high-density human mitochondrial proximity interaction network. *Cell Metab.* 32 479–497.e9. 32877691 10.1016/j.cmet.2020.07.017

[B6] Arruda A. P. Pers B. M. Parlakgul G. Guney E. Inouye K. Hotamisligil G. S. (2014). Chronic enrichment of hepatic endoplasmic reticulum-mitochondria contact leads to mitochondrial dysfunction in obesity. *Nat. Med.* 20 1427–1435. 10.1038/nm.3735 25419710 PMC4412031

[B7] Axe E. L. Walker S. A. Manifava M. Chandra P. Roderick H. L. Habermann A. (2008). Autophagosome formation from membrane compartments enriched in phosphatidylinositol 3-phosphate and dynamically connected to the endoplasmic reticulum. *J. Cell Biol.* 182 685–701. 10.1083/jcb.200803137 18725538 PMC2518708

[B8] Baird D. Stefan C. Audhya A. Weys S. Emr S. D. (2008). Assembly of the PtdIns 4-kinase Stt4 complex at the plasma membrane requires Ypp1 and Efr3. *J. Cell Biol.* 183 1061–1074. 10.1083/jcb.200804003 19075114 PMC2600738

[B9] Balla T. Kim Y. J. Alvarez-Prats A. Pemberton J. (2019). Lipid dynamics at contact sites between the endoplasmic reticulum and other organelles. *Annu. Rev. Cell Dev. Biol.* 35 85–109. 10.1146/annurev-cellbio-100818-125251 31590585

[B10] Balla T. Sengupta N. Kim Y. J. (2020). Lipid synthesis and transport are coupled to regulate membrane lipid dynamics in the endoplasmic reticulum. *Biochim. Biophys. Acta Mol. Cell Biol. Lipids* 1865 158461. 10.1016/j.bbalip.2019.05.005 31108203 PMC6858525

[B11] Barbosa A. D. Sembongi H. Su W. M. Abreu S. Reggiori F. Carman G. M. (2015). Lipid partitioning at the nuclear envelope controls membrane biogenesis. *Mol. Biol. Cell* 26 3641–3657. 10.1091/mbc.e15-03-0173 26269581 PMC4603934

[B12] Benador I. Y. Veliova M. Mahdaviani K. Petcherski A. Wikstrom J. D. Assali E. A. (2018). Mitochondria bound to lipid droplets have unique bioenergetics, composition, and dynamics that support lipid droplet expansion. *Cell Metab.* 27 869–885.e6. 29617645 10.1016/j.cmet.2018.03.003 PMC5969538

[B13] Castellano B. M. Thelen A. M. Moldavski O. Feltes M. van der Welle R. E. N. Mydock-McGrane L. (2017). CHOLESTEROL SENSING Lysosomal cholesterol activates mTORC1 via an SLC38A9-Niemann-Pick C1 signaling complex. *Science* 355 1306–1311. 10.1126/science.aag1417 28336668 PMC5823611

[B14] Chen Y. F. Kao C. H. Chen Y. T. Wang C. H. Wu C. Y. Tsai C. Y. (2009). Cisd2 deficiency drives premature aging and causes mitochondria-mediated defects in mice. *Gene Dev.* 23 1183–1194. 10.1101/gad.1779509 19451219 PMC2685531

[B15] Cho I. T. Adelmant G. Lim Y. Marto J. A. Cho G. Golden J. A. (2017). Ascorbate peroxidase proximity labeling coupled with biochemical fractionation identifies promoters of endoplasmic reticulum-mitochondrial contacts. *J. Biol. Chem.* 292 16382–16392. 10.1074/jbc.m117.795286 28760823 PMC5625067

[B16] Cui Z. Vance J. E. Chen M. H. Voelker D. R. Vance D. E. (1993). Cloning and expression of a novel phosphatidylethanolamine N-methyltransferase - a specific biochemical and cytological marker for a unique membrane-fraction in rat-liver. *J. Biol. Chem.* 268 16655–16663. 8344945

[B17] D’Angelo G. Vicinanza M. Di Campli A. De Matteis M. A. (2008). The multiple roles of PtdIns(4)P - not just the precursor of PtdIns(4,5)P-2. *J. Cell. Sci.* 121 1955–1963. 10.1242/jcs.023630 18525025

[B18] Datta S. Liu Y. Hariri H. Bowerman J. Henne W. M. (2019). Cerebellar ataxia disease-associated Snx14 promotes lipid droplet growth at ER-droplet contacts. *J. Cell Biol.* 218 1335–1351. 10.1083/jcb.201808133 30765438 PMC6446855

[B19] Dickson E. J. Jensen J. B. Vivas O. Kruse M. Traynor-Kaplan A. E. Hille B. (2016). Dynamic formation of ER-PM junctions presents a lipid phosphatase to regulate phosphoinositides. *J. Cell Biol.* 213 33–48. 10.1083/jcb.201508106 27044890 PMC4828688

[B20] Doghman-Bouguerra M. Granatiero V. Sbiera S. Sbiera I. Lacas-Gervais S. Brau F. (2016). FATE1 antagonizes calcium- and drug-induced apoptosis by uncoupling ER and mitochondria. *EMBO Rep.* 17 1264–1280. 10.15252/embr.201541504 27402544 PMC5007562

[B21] Eisenberg-Bord M. Mari M. Weill U. Rosenfeld-Gur E. Moldavski O. Castro I. G. (2018). Identification of seipin-linked factors that act as determinants of a lipid droplet subpopulation. *J. Cell Biol.* 217 269–282. 10.1083/jcb.201704122 29187527 PMC5748981

[B22] Fan J. J. Li X. L. Issop L. Culty M. Papadopoulos V. (2016). ACBD2/ECI2-mediated peroxisome-mitochondria interactions in leydig cell steroid biosynthesis. *Mol. Endocrinol.* 30 763–782. 10.1210/me.2016-1008 27167610 PMC5426581

[B23] Faulhammer F. Kanjilal-Kolar S. Knodler A. Lo J. Lee Y. Konrad G. (2007). Growth control of golgi phosphoinositides by reciprocal localization of sac1 lipid phosphatase and pik1 4-kinase. *Traffic* 8 1554–1567. 10.1111/j.1600-0854.2007.00632.x 17908202

[B24] Fernandez-Murray J. P. McMaster C. R. (2016). Lipid synthesis and membrane contact sites: a crossroads for cellular physiology. *J. Lipid Res.* 57 1789–1805. 10.1194/jlr.r070920 27521373 PMC5036376

[B25] Fischl A. S. Carman G. M. (1983). Phosphatidylinositol biosynthesis in *Saccharomyces cerevisiae*: purification and properties of microsome-associated phosphatidylinositol synthase. *J. Bacteriol.* 154 304–311. 10.1128/jb.154.1.304-311.1983 6300035 PMC217460

[B26] Foti M. Audhya A. Emr S. D. (2001). Sac1 lipid phosphatase and Stt4 phosphatidylinositol 4-kinase regulate a pool of phosphatidylinositol 4-phosphate that functions in the control of the actin cytoskeleton and vacuole morphology. *Mol. Biol. Cell* 12 2396–2411. 10.1091/mbc.12.8.2396 11514624 PMC58602

[B27] Freyre C. A. C. Rauher P. C. Ejsing C. S. Klemm R. W. (2019). MIGA2 links mitochondria, the ER, and lipid droplets and promotes *de novo* lipogenesis in adipocytes. *Mol. Cell* 76 811–825.e14. 31628041 10.1016/j.molcel.2019.09.011

[B28] Friedman J. R. Kannan M. Toulmay A. Jan C. H. Weissman J. S. Prinz W. A. (2018). Lipid homeostasis is maintained by dual targeting of the mitochondrial PE biosynthesis enzyme to the ER. *Dev. Cell* 44 261–270.e6. 29290583 10.1016/j.devcel.2017.11.023 PMC5975648

[B29] Fu S. N. Yang L. Li P. Hofmann O. Dicker L. Hide W. (2011). Aberrant lipid metabolism disrupts calcium homeostasis causing liver endoplasmic reticulum stress in obesity. *Nature* 473 528–531. 10.1038/nature09968 21532591 PMC3102791

[B30] Gaigg B. Simbeni R. Hrastnik C. Paltauf F. Daum G. (1995). Characterization of a microsomal subfraction associated with mitochondria of the yeast, *Saccharomyces cerevisiae*. Involvement in synthesis and import of phospholipids into mitochondria. *Biochim. Biophys. Acta* 1234 214–220. 10.1016/0005-2736(94)00287-y 7696296

[B31] Garofalo T. Matarrese P. Manganelli V. Marconi M. Tinari A. Gambardella L. (2016). Evidence for the involvement of lipid rafts localized at the ER-mitochondria associated membranes in autophagosome formation. *Autophagy* 12 917–935. 10.1080/15548627.2016.1160971 27123544 PMC4922444

[B32] Geng J. F. Nair U. Yasumura-Yorimitsu K. Klionsky D. J. (2010). Post-Golgi sec proteins are required for autophagy in *Saccharomyces cerevisiae*. *Mol. Biol. Cell* 21 2257–2269. 10.1091/mbc.e09-11-0969 20444978 PMC2893989

[B33] Gong X. Qian H. W. Zhou X. H. Wu J. P. Wan T. Cao P. P. (2016). Structural insights into the Niemann-Pick C1 (NPC1)-mediated cholesterol transfer and ebola infection. *Cell* 165 1467–1478. 10.1016/j.cell.2016.05.022 27238017 PMC7111323

[B34] Gonzalez-Baro M. R. Lewin T. M. Coleman R. A. (2007). Regulation of triglyceride metabolism II. Function of mitochondrial GPAT1 in the regulation of triacylglycerol biosynthesis and insulin action. *Am. J. Physiol. Gastrointest. Liver Physiol.* 292 G1195–G1199. 17158253 10.1152/ajpgi.00553.2006 PMC2819211

[B35] Gutierrez T. Parra V. Troncoso R. Pennanen C. Contreras-Ferrat A. Vasquez-Trincado C. (2014). Alteration in mitochondrial Ca2+ uptake disrupts insulin signaling in hypertrophic cardiomyocytes. *Cell Commun. Signal.* 12:68 10.1186/preaccept-1950166084128344 PMC4234850 25376904

[B36] Hailey D. W. Rambold A. S. Satpute-Krishnan P. Mitra K. Sougrat R. Kim P. K. (2010). Mitochondria supply membranes for autophagosome biogenesis during starvation. *Cell* 141 656–667. 10.1016/j.cell.2010.04.009 20478256 PMC3059894

[B37] Harder T. Simons K. (1997). Caveolae, DIGs, and the dynamics of sphingolipid-cholesterol microdomains. *Curr. Opin. Cell Biol.* 9 534–542. 10.1016/s0955-0674(97)80030-0 9261060

[B38] Hariri H. Rogers S. Ugrankar R. Liu Y. L. Feathers J. R. Henne W. M. (2018). Lipid droplet biogenesis is spatially coordinated at ER-vacuole contacts under nutritional stress. *EMBO Rep.* 19 57–72. 10.15252/embr.201744815 29146766 PMC5757283

[B39] Hayashi T. Fujimoto M. (2010). Detergent-resistant microdomains determine the localization of sigma-1 receptors to the endoplasmic reticulum-mitochondria junction. *Mol. Pharmacol.* 77 517–528. 10.1124/mol.109.062539 20053954 PMC2845942

[B40] Hayashi T. Su T. P. (2007). Sigma-1 receptor chaperones at the ER-mitochondrion interface regulate Ca(2+) signaling and cell survival. *Cell* 131 596–610. 10.1016/j.cell.2007.08.036 17981125

[B41] Hernandez-Alvarez M. I. Sebastian D. Vives S. Ivanova S. Bartoccioni P. Kakimoto P. (2019). Deficient endoplasmic reticulum-mitochondrial phosphatidylserine transfer causes liver disease. *Cell* 177 881–895.e17. 31051106 10.1016/j.cell.2019.04.010

[B42] Horvath S. E. Daum G. (2013). Lipids of mitochondria. *Prog. Lipid Res.* 52 590–614. 24007978 10.1016/j.plipres.2013.07.002

[B43] Hung V. Lam S. S. Udeshi N. D. Svinkina T. Guzman G. Mootha V. K. (2017). Proteomic mapping of cytosol-facing outer mitochondrial and ER membranes in living human cells by proximity biotinylation. *eLife* 6:e24463. 10.7554/eLife.24463 PMC5404927 28441135

[B44] Jacobs R. L. Jiang H. Kennelly J. P. Orlicky D. J. Allen R. H. Stabler S. P. (2017). Cystathionine beta-synthase deficiency alters hepatic phospholipid and choline metabolism: post-translational repression of phosphatidylethanolamine N-methyltransferase is a consequence rather than a cause of liver injury in homocystinuria. *Mol. Genet. Metab.* 120 325–336. 10.1016/j.ymgme.2017.02.010 28291718

[B45] Kannan M. Lahiri S. Liu L. K. Choudhary V. Prinz W. A. (2017). Phosphatidylserine synthesis at membrane contact sites promotes its transport out of the ER. *J. Lipid Res.* 58 553–562. 10.1194/jlr.m072959 28119445 PMC5335585

[B46] Karanasios E. Barbosa A. D. Sembongi H. Mari M. Han G. S. Reggiori F. (2013). Regulation of lipid droplet and membrane biogenesis by the acidic tail of the phosphatidate phosphatase Pah1p. *Mol. Biol. Cell* 24 2124–2133. 10.1091/mbc.e13-01-0021 23657815 PMC3694796

[B47] Kawano S. Tamura Y. Kojima R. Bala S. Asai E. Michel A. H. (2018). Structure-function insights into direct lipid transfer between membranes by Mmm1-Mdm12 of ERMES. *J. Cell Biol.* 217, 959–974. 10.1083/jcb.201704119 29279306 PMC5839780

[B48] Kim Y. J. Guzman-Hernandez M. L. Balla T. (2011). A highly dynamic ER-derived phosphatidylinositol-synthesizing organelle supplies phosphoinositides to cellular membranes. *Dev. Cell* 21 813–824. 10.1016/j.devcel.2011.09.005 22075145 PMC3235737

[B49] King C. Sengupta P. Seo A. Y. Lippincott-Schwartz J. (2020). ER membranes exhibit phase behavior at sites of organelle contact. *Proc. Natl. Acad. Sci. U.S.A.* 117 7225–7235. 10.1073/pnas.1910854117 32179693 PMC7132286

[B50] Kong J. Ji Y. Jeon Y. G. Han J. S. Han K. H. Lee J. H. (2020). Spatiotemporal contact between peroxisomes and lipid droplets regulates fasting-induced lipolysis via PEX5. *Nat. Commun.* 11:578. 10.1038/s41467-019-14176-0 PMC6989686 31996685

[B51] Kwak C. Shin S. Park J. S. Jung M. Nhung T. T. M. Kang M. G. (2020). Contact-ID, a tool for profiling organelle contact sites, reveals regulatory proteins of mitochondrial-associated membrane formation. *Proc. Natl. Acad. Sci. U.S.A.* 117 12109–12120. 10.1073/pnas.1916584117 32414919 PMC7275737

[B52] Lahiri S. Chao J. T. Tavassoli S. Wong A. K. Choudhary V. Young B. P. (2014). A conserved endoplasmic reticulum membrane protein complex (EMC) facilitates phospholipid transfer from the ER to mitochondria. *PLoS Biol.* 12:e1001969. 10.1371/journal.pbio.1001969 25313861 PMC4196738

[B53] Lahiri S. Toulmay A. Prinz W. A. (2015). Membrane contact sites, gateways for lipid homeostasis. *Curr. Opin. Cell Biol.* 33 82–87. 10.1016/j.ceb.2014.12.004 25569848 PMC4380522

[B54] Layerenza J. P. Gonzalez P. de Bravo M. M. G. Polo M. P. Sisti M. S. Ves-Losada A. (2013). Nuclear lipid droplets: a novel nuclear domain. *Biochim. Biophys. Acta* 1831 327–340. 10.1016/j.bbalip.2012.10.005 23098923

[B55] Lees J. A. Messa M. Sun E. W. Wheeler H. Torta F. Wenk M. R. (2017). Lipid transport by TMEM24 at ER-plasma membrane contacts regulates pulsatile insulin secretion. *Science* 355 eaah6171. 10.1126/science.aah6171 28209843 PMC5414417

[B56] Lim C. Y. Davis O. B. Shin H. R. Zhang J. Berdan C. A. Jiang X. T. (2019). ER-lysosome contacts enable cholesterol sensing by mTORC1 and drive aberrant growth signalling in Niemann-Pick type C. *Nat. Cell Biol.* 21 1206–1218. 10.1038/s41556-019-0391-5 31548609 PMC6936960

[B57] Ma J. H. J. Shen S. C. Wang J. J. He Z. W. Poon A. Li J. (2017). Comparative proteomic analysis of the mitochondria-associated ER Membrane (MAM) in a Long-term Type 2 diabetic rodent model. *Sci. Rep.* 7:2062. 10.1038/s41598-017-02213-1 PMC5437025 28522876

[B58] Mari M. Griffith J. Rieter E. Krishnappa L. Klionsky D. J. Reggiori F. (2010). An Atg9-containing compartment that functions in the early steps of autophagosome biogenesis. *J. Cell Biol.* 190 1005–1022. 10.1083/jcb.200912089 20855505 PMC3101592

[B59] McDonough V. M. Buxeda R. J. Bruno M. E. Ozier-Kalogeropoulos O. Adeline M. T. McMaster C. R. (1995). Regulation of phospholipid biosynthesis in *Saccharomyces cerevisiae* by CTP. *J. Biol. Chem.* 270 18774–18780. 7642527 10.1074/jbc.270.32.18774

[B60] Mesmin B. Bigay J. Moser von Filseck J. Lacas-Gervais S. Drin G. Antonny B. (2013). A four-step cycle driven by PI(4)P hydrolysis directs sterol/PI(4)P exchange by the ER-Golgi tether OSBP. *Cell* 155 830–843. 10.1016/j.cell.2013.09.056 24209621

[B61] Mesmin B. Bigay J. Polidori J. Jamecna D. Lacas-Gervais S. Antonny B. (2017). Sterol transfer, PI4P consumption, and control of membrane lipid order by endogenous OSBP. *EMBO J.* 36 3156–3174. 10.15252/embj.201796687 28978670 PMC5666618

[B62] Muallem S. Chung W. Y. Jha A. Ahuja M. (2017). Lipids at membrane contact sites: cell signaling and ion transport. *EMBO Rep.* 18 1893–1904. 10.15252/embr.201744331 29030479 PMC5666614

[B63] Murley A. Sarsam R. D. Toulmay A. Yamada J. Prinz W. A. Nunnari J. (2015). Ltc1 is an ER-localized sterol transporter and a component of ER-mitochondria and ER-vacuole contacts. *J. Cell Biol.* 209 539–548. 10.1083/jcb.201502033 25987606 PMC4442815

[B64] Murley A. Yamada J. Niles B. J. Toulmay A. Prinz W. A. Powers T. (2017). Sterol transporters at membrane contact sites regulate TORC1 and TORC2 signaling. *J. Cell Biol.* 216 2679–2689. 10.1083/jcb.201610032 28774891 PMC5584152

[B65] Nagle C. A. Vergnes L. Dejong H. Wang S. Lewin T. M. Reue K. (2008). Identification of a novel sn-glycerol-3-phosphate acyltransferase isoform, GPAT4, as the enzyme deficient in Agpat6-/- mice. *J. Lipid Res.* 49 823–831. 10.1194/jlr.m700592-jlr200 18192653 PMC2819352

[B66] Nemoto Y. Kearns B. G. Wenk M. R. Chen H. Mori K. Alb J. G. (2000). Functional characterization of a mammalian Sac1 and mutants exhibiting substrate-specific defects in phosphoinositide phosphatase activity. *J. Biol. Chem.* 275 34293–34305. 10.1074/jbc.m003923200 10887188

[B67] Nguyen T. T. Lewandowska A. Choi J. Y. Markgraf D. F. Junker M. Bilgin M. (2012). Gem1 and ERMES do not directly affect phosphatidylserine transport from ER to mitochondria or mitochondrial inheritance. *Traffic* 13 880–890. 10.1111/j.1600-0854.2012.01352.x 22409400 PMC3648210

[B68] Nishimura T. Gecht M. Covino R. Hummer G. Surma M. A. Klose C. (2019). Osh proteins control nanoscale lipid organization necessary for PI(4,5)P-2 synthesis. *Mol. Cell* 75 1043–1057.e8. 31402097 10.1016/j.molcel.2019.06.037 PMC6739424

[B69] Nishimura T. Tamura N. Kono N. Shimanaka Y. Arai H. Yamamoto H. (2017). Autophagosome formation is initiated at phosphatidylinositol synthase-enriched ER subdomains. *EMBO J.* 36 1719–1735. 10.15252/embj.201695189 28495679 PMC5470044

[B70] Ohsaki Y. Kawai T. Yoshikawa Y. Cheng J. Jokitalo E. Fujimoto T. (2016). PML isoform II plays a critical role in nuclear lipid droplet formation. *J. Cell Biol.* 212 29–38. 10.1083/jcb.201507122 26728854 PMC4700481

[B71] Pellon-Malson M. Montanaro M. A. Coleman R. A. Gonzalez-Baro M. R. (2007). Mitochondrial glycerol-3-P acyltransferase 1 is most active in outer mitochondrial membrane but not in mitochondrial associated vesicles (MAV). *Biochim. Biophys. Acta* 1771 830–838. 10.1016/j.bbalip.2007.04.001 17493869 PMC2230616

[B72] Petrungaro C. Kornmann B. (2019). Lipid exchange at ER-mitochondria contact sites: a puzzle falling into place with quite a few pieces missing. *Curr. Opin. Cell Biol.* 57 71–76. 10.1016/j.ceb.2018.11.005 30554079

[B73] Phillips M. J. Voeltz G. K. (2016). Structure and function of ER membrane contact sites with other organelles. *Nat. Rev. Mol. Cell Biol.* 17 69–82. 10.1038/nrm.2015.8 26627931 PMC5117888

[B74] Pichler H. Gaigg B. Hrastnik C. Achleitner G. Kohlwein S. D. Zellnig G. (2001). A subfraction of the yeast endoplasmic reticulum associates with the plasma membrane and has a high capacity to synthesize lipids. *Eur. J. Biochem.* 268 2351–2361. 10.1046/j.1432-1327.2001.02116.x 11298754

[B75] Poston C. N. Duong E. Cao Y. Bazemore-Walker C. R. (2011). Proteomic analysis of lipid raft-enriched membranes isolated from internal organelles. *Biochem. Biophys. Res. Commun.* 415 355–360. 10.1016/j.bbrc.2011.10.072 22037461 PMC3253365

[B76] Prinz W. A. Toulmay A. Balla T. (2020). The functional universe of membrane contact sites. *Nat. Rev. Mol. Cell Biol.* 21 7–24. 10.1038/s41580-019-0180-9 31732717 PMC10619483

[B77] Puri C. Renna M. Bento C. F. Moreau K. Rubinsztein D. C. (2013). Diverse autophagosome membrane sources coalesce in recycling endosomes. *Cell* 154 1285–1299. 10.1016/j.cell.2013.08.044 24034251 PMC3791395

[B78] Quon E. Sere Y. Y. Chauhan N. Johansen J. Sullivan D. P. Dittman J. S. (2018). Endoplasmic reticulum-plasma membrane contact sites integrate sterol and phospholipid regulation. *PLoS Biol.* 16:e2003864. 10.1371/journal.pbio.2003864 29782498 PMC5983861

[B79] Rai A. Pathak D. Thakur S. Singh S. Dubey A. K. Mallik R. (2016). Dynein clusters into lipid microdomains on phagosomes to drive rapid transport toward lysosomes. *Cell* 164 722–734. 10.1016/j.cell.2015.12.054 26853472 PMC4752818

[B80] Rusinol A. E. Cui Z. Chen M. H. Vance J. E. (1994). A Unique mitochondria-associated membrane-fraction from rat-liver has a high-capacity for lipid-synthesis and contains pre-golgi secretory proteins including nascent lipoproteins. *J. Biol. Chem.* 269 27494–27502. 7961664

[B81] Sala-Vila A. Navarro-Lerida I. Sanchez-Alvarez M. Bosch M. Calvo C. Lopez J. A. (2016). Interplay between hepatic mitochondria-associated membranes, lipid metabolism and caveolin-1 in mice. *Sci. Rep.* 6:27351. 10.1038/srep27351 PMC4894368 27272971

[B82] Sano R. Annunziata I. Patterson A. Moshiach S. Gomero E. Opferman J. (2009). GM1-ganglioside accumulation at the mitochondria-associated ER membranes links ER stress to Ca2+-dependent mitochondrial apoptosis. *Mol. Cell* 36 500–511. 10.1016/j.molcel.2009.10.021 19917257 PMC2782904

[B83] Schueller C. Mamnun Y. M. Wolfger H. Rockwell N. Thorner J. Kuchler K. (2007). Membrane-active compounds activate the transcription factors Pdr1 and Pdr3 connecting pleiotropic drug resistance and membrane lipid homeostasis in *Saccharomyces cerevisiae*. *Mol. Biol. Cell* 18 4932–4944. 10.1091/mbc.e07-06-0610 17881724 PMC2096591

[B84] Schuiki I. Schnabl M. Czabany T. Hrastnik C. Daum G. (2010). Phosphatidylethanolamine synthesized by four different pathways is supplied to the plasma membrane of the yeast *Saccharomyces cerevisiae*. *Biochim. Biophys. Acta* 1801 480–486. 10.1016/j.bbalip.2009.12.008 20044027

[B85] Schuldiner M. Bohnert M. (2017). A different kind of love - lipid droplet contact sites. *Biochim. Biophys. Acta* 1862 1188–1196. 10.1016/j.bbalip.2017.06.005 28627434

[B86] Schutter M. Giavalisco P. Brodesser S. Graef M. (2020). Local fatty acid channeling into phospholipid synthesis drives phagophore expansion during autophagy. *Cell* 180 135–149.e14. 31883797 10.1016/j.cell.2019.12.005

[B87] Scorrano L. De Matteis M. A. Emr S. Giordano F. Hajnoczky G. Kornmann B. (2019). Coming together to define membrane contact sites. *Nat. Commun.* 10:1287. 10.1038/s41467-019-09253-3 PMC6427007 30894536

[B88] Sebastian D. Hernandez-Alvarez M. I. Segales J. Sorianello E. Munoz J. P. Sala D. (2012). Mitofusin 2 (Mfn2) links mitochondrial and endoplasmic reticulum function with insulin signaling and is essential for normal glucose homeostasis. *Proc. Natl. Acad. Sci. U.S.A.* 109 5523–5528. 10.1073/pnas.1108220109 22427360 PMC3325712

[B89] Shai N. Yifrach E. van Roermund C. W. T. Cohen N. Bibi C. Ijlst L. (2018). Systematic mapping of contact sites reveals tethers and a function for the peroxisome-mitochondria contact. *Nat. Commun.* 9:1761. 10.1038/s41467-018-03957-8 PMC5932058 29720625

[B90] Shiao Y. J. Lupo G. Vance J. E. (1995). Evidence that phosphatidylserine is imported into mitochondria via a mitochondria-associated membrane and that the majority of mitochondrial phosphatidylethanolamine is derived from decarboxylation of phosphatidylserine. *J. Biol. Chem.* 270 11190–11198. 10.1074/jbc.270.19.11190 7744750

[B91] Shima T. Kirisako H. Nakatogawa H. (2019). COPII vesicles contribute to autophagosomal membranes. *J. Cell Biol.* 218 1503–1510. 10.1083/jcb.201809032 30787039 PMC6504894

[B92] Shinjo S. Jiang S. Y. Nameta M. Suzuki T. Kanai M. Nomura Y. (2017). Disruption of the mitochondria-associated ER membrane (MAM) plays a central role in palmitic acid induced insulin resistance. *Exp. Cell Res.* 359 86–93. 10.1016/j.yexcr.2017.08.006 28827061

[B93] Simmen T. Herrera-Cruz M. S. (2018). Plastic mitochondria-endoplasmic reticulum (ER) contacts use chaperones and tethers to mould their structure and signaling. *Curr. Opin. Cell Biol.* 53 61–69. 10.1016/j.ceb.2018.04.014 29870872

[B94] Simons K. Ikonen E. (1997). Functional rafts in cell membranes. *Nature* 387 569–572. 10.1038/42408 9177342

[B95] Stefan C. J. Manford A. G. Baird D. Yamada-Hanff J. Mao Y. X. Emr S. D. (2011). Osh proteins regulate phosphoinositide metabolism at ER-plasma membrane contact sites. *Cell* 144 389–401. 10.1016/j.cell.2010.12.034 21295699

[B96] Stone S. J. Vance J. E. (2000). Phosphatidylserine synthase-1 and-2 are localized to mitochondria-associated membranes. *J. Biol. Chem.* 275 34534–34540. 10.1074/jbc.m002865200 10938271

[B97] Suresh H. G. dos Santos A. X. D. Kukulski W. Tyedmers J. Riezman H. Bukau B. (2015). Prolonged starvation drives reversible sequestration of lipid biosynthetic enzymes and organelle reorganization in *Saccharomyces cerevisiae*. *Mol. Biol. Cell* 26 1601–1615. 10.1091/mbc.e14-11-1559 25761633 PMC4436773

[B98] Takeuchi K. Reue K. (2009). Biochemistry, physiology, and genetics of GPAT, AGPAT, and lipin enzymes in triglyceride synthesis. *Am. J. Physiol. Endocrinol. Metab.* 296 E1195–E1209. 19336658 10.1152/ajpendo.90958.2008 PMC2692402

[B99] Tamura Y. Kawano S. Endo T. (2019). Organelle contact zones as sites for lipid transfer. *J. Biochem.* 165 115–123. 10.1093/jb/mvy088 30371789

[B100] Tavassoli S. Chao J. T. Young B. P. Cox R. C. Prinz W. A. de Kroon A. I. P. M. (2013). Plasma membrane-endoplasmic reticulum contact sites regulate phosphatidylcholine synthesis. *EMBO Rep.* 14 434–440. 10.1038/embor.2013.36 23519169 PMC3642376

[B101] Theurey P. Tubbs E. Vial G. Jacquemetton J. Bendridi N. Chauvin M. A. (2016). Mitochondria-associated endoplasmic reticulum membranes allow adaptation of mitochondrial metabolism to glucose availability in the liver. *J. Mol. Cell. Biol.* 8 129–143. 10.1093/jmcb/mjw004 26892023

[B102] Thoudam T. Ha C. M. Leem J. Chanda D. Park J. S. Kim H. J. (2019). PDK4 Augments ER-mitochondria contact to dampen skeletal muscle insulin signaling during obesity. *Diabetes* 68 571–586. 10.2337/db18-0363 30523025 PMC6385748

[B103] Tubbs E. Chanon S. Robert M. Bendridi N. Bidaux G. Chauvin M. A. (2018). Disruption of mitochondria-associated endoplasmic reticulum membrane (MAM) Integrity contributes to muscle insulin resistance in mice and humans. *Diabetes* 67 636–650. 10.2337/db17-0316 29326365

[B104] Tubbs E. Theurey P. Vial G. Bendridi N. Bravard A. Chauvin M. A. (2014). Mitochondria-associated endoplasmic reticulum membrane (MAM) integrity is required for insulin signaling and is implicated in hepatic insulin resistance. *Diabetes* 63 3279–3294. 10.2337/db13-1751 24947355

[B105] Uzbekov R. Roingeard P. (2013). Nuclear lipid droplets identified by electron microscopy of serial sections. *BMC Res. Notes* 6:386. 10.1186/1756-0500-6-386 24070407 PMC3849021

[B106] van Meer G. Voelker D. R. Feigenson G. W. (2008). Membrane lipids: where they are and how they behave. *Nat. Rev. Mol. Cell Biol.* 9 112–124. 10.1038/nrm2330 18216768 PMC2642958

[B107] Vance J. E. (1990). Phospholipid synthesis in a membrane fraction associated with mitochondria. *J. Biol. Chem.* 265 7248–7256. 2332429

[B108] Vance J. E. (1991). Newly made phosphatidylserine and phosphatidylethanolamine are preferentially translocated between rat liver mitochondria and endoplasmic reticulum. *J. Biol. Chem.* 266 89–97. 1898727

[B109] Vance J. E. Vance D. E. (1988). Does rat-liver golgi have the capacity to synthesize phospholipids for lipoprotein secretion. *J. Biol. Chem.* 263 5898–5909. 2833521

[B110] Vidugiriene J. Sharma D. K. Smith T. K. Baumann N. A. Menon A. K. (1999). Segregation of glycosylphosphatidylinositol biosynthetic reactions in a subcompartment of the endoplasmic reticulum. *J. Biol. Chem.* 274 15203–15212. 10.1074/jbc.274.21.15203 10329729

[B111] Voelker D. R. (1990). Characterization of phosphatidylserine synthesis and translocation in permeabilized animal-cells. *J. Biol. Chem.* 265 14340–14346. 2117609

[B112] von Filseck J. M. Copic A. Delfosse V. Vanni S. Jackson C. L. Bourguet W. (2015). Phosphatidylserine transport by ORP/Osh proteins is driven by phosphatidylinositol 4-phosphate. *Science* 349 432–436. 10.1126/science.aab1346 26206936

[B113] Wang C. H. Chen Y. F. Wu C. Y. Wu P. C. Huang Y. L. Kao C. H. (2014). Cisd2 modulates the differentiation and functioning of adipocytes by regulating intracellular Ca2+ homeostasis. *Hum. Mol. Genet.* 23 4770–4785. 10.1093/hmg/ddu193 24833725

[B114] Wang S. Lee D. P. Gong N. Schwerbrock N. M. Mashek D. G. Gonzalez-Baro M. R. (2007). Cloning and functional characterization of a novel mitochondrial N-ethylmaleimide-sensitive glycerol-3-phosphate acyltransferase (GPAT2). *Arch. Biochem. Biophys.* 465 347–358. 10.1016/j.abb.2007.06.033 17689486 PMC2133398

[B115] Wang X. L. Wen Y. J. Dong J. Cao C. C. Yuan S. Q. (2018). Systematic in-depth proteomic analysis of mitochondria-associated endoplasmic reticulum membranes in mouse and human testes. *Proteomics* 18:e1700478. 10.1002/pmic.201700478 29785746

[B116] Wang Y. Yuan P. Grabon A. Tripathi A. Lee D. Rodriguez M. (2020). Noncanonical regulation of phosphatidylserine metabolism by a Sec14-like protein and a lipid kinase. *J. Cell Biol.* 219:e201907128. 10.1083/jcb.201907128 PMC7199851 32303746

[B117] Wu S. Lu Q. Ding Y. Wu Y. Qiu Y. Wang P. (2019). Hyperglycemia-driven inhibition of AMP-activated protein kinase alpha2 induces diabetic cardiomyopathy by promoting mitochondria-associated endoplasmic reticulum membranes *in vivo*. *Circulation* 139 1913–1936. 10.1161/circulationaha.118.033552 30646747 PMC6465113

[B118] Xie B. Nguyen P. M. Gucek A. Thonig A. Barg S. Idevall-Hagren O. (2016). Plasma membrane phosphatidylinositol 4,5-bisphosphate regulates Ca(2+)-Influx and Insulin Secretion from Pancreatic beta Cells. *Cell Chem. Biol.* 23 816–826. 10.1016/j.chembiol.2016.06.009 27447049

[B119] Zaman S. Lippman S. I. Zhao X. Broach J. R. (2008). How *Saccharomyces* responds to nutrients. *Annu. Rev. Genet.* 42 27–81. 10.14492/hokmj/1381758488 18303986

[B120] Zhao Y. G. Zhang H. (2019). Autophagosome maturation: an epic journey from the ER to lysosomes. *J. Cell Biol.* 218 757–770. 10.1083/jcb.201810099 30578282 PMC6400552

